# Circadian rhythm of exhaled biomarkers in health and asthma

**DOI:** 10.1183/13993003.01068-2019

**Published:** 2019-10-17

**Authors:** Maxim Wilkinson, Robert Maidstone, Andrew Loudon, John Blaikley, Iain R. White, Dave Singh, David W. Ray, Royston Goodacre, Stephen J. Fowler, Hannah J. Durrington

**Affiliations:** 1 Division of Infection, Immunity and Respiratory Medicine, School of Biological Sciences, Faculty of Biology, Medicine and Health, University of Manchester, Manchester, UK; 2 NIHR Oxford Biomedical Research Centre, John Radcliffe Hospital, Oxford, UK; 3 Oxford Centre for Diabetes, Endocrinology and Metabolism, University of Oxford, Oxford, UK; 4 Division of Informatics, Imaging and Data Sciences, School of Biological Sciences, Faculty of Biology, Medicine and Health, University of Manchester, Manchester, UK; 5 Division of Diabetes, Endocrinology and Gastroenterology, School of Medical Sciences, Faculty of Biology, Medicine and Health, University of Manchester, Manchester, UK; 6 Manchester Academic Health Science Centre and NIHR Biomedical Research Centre, Manchester University Hospitals NHS Foundation Trust, Manchester, UK; 7 Laboratory for Environmental and Life Sciences, University of Nova Gorica, Nova Gorica, Slovenia; 8 Medicines Evaluation Unit (MEU), Langley Building, Manchester University Hospitals NHS Foundation Trust, Manchester, UK; 9 School of Chemistry, Manchester Institute of Biotechnology, The University of Manchester, Manchester, UK; 10 Dept of Biochemistry, Institute of Integrative Biology, University of Liverpool, Liverpool, UK

## Abstract

Circadian rhythms regulate and reflect many biological processes. Investigating circadian variability in biomarkers is important since the diurnal variability of any potential biomarker must be quantified and controlled in research and clinical practice. Time of day is particularly important in inflammatory diseases such as asthma, which are linked to exaggerated circadian rhythms. Airway narrowing in asthma is greatest at around 04:00 h and coincides with an increase in symptoms; asthma deaths are also more likely to occur at this time [1, 2]. Likewise eosinophilic airway inflammation peaks in the morning, with clinical implications for biomarker-guided steroid therapy [3].

## Abstract 2

**Exhaled volatile chemicals and fractional exhaled nitric oxide oscillate over 24 h, highlighting the importance of time of day in diagnostic sampling and suggesting potential applications for chronotyping.** http://bit.ly/2YZGXbH

To the Editor:

Circadian rhythms regulate and reflect many biological processes. Investigating circadian variability in biomarkers is important since the diurnal variability of any potential biomarker must be quantified and controlled in research and clinical practice. Time of day is particularly important in inflammatory diseases such as asthma, which are linked to exaggerated circadian rhythms. Airway narrowing in asthma is greatest at around 04:00 h and coincides with an increase in symptoms; asthma deaths are also more likely to occur at this time [1, 2]. Likewise eosinophilic airway inflammation peaks in the morning, with clinical implications for biomarker-guided steroid therapy [3].

As asthma is a circadian disease, we expected to observe newly rhythmic volatile organic compounds (VOCs) in breath when compared to a healthy population. Therefore, we investigated how exhaled VOCs and exhaled nitric oxide fraction (*F*_eNO_) vary over the 24-h cycle in healthy individuals and in those with asthma.

During an overnight visit to the research unit, exhaled breath was collected and *F*_eNO_ was measured at 16:00 h, 22:00 h, 04:00 h and 10:00 h. Participants took standardised meals at regular intervals and kept their usual bedtime. Inhaled corticosteroids (ICS) were omitted 12 h prior to measurements. The study protocol received ethical approval (ref: 14/NW/1352) and participants provided written informed consent.

*F*_eNO_ measurements were performed (NIOX Vero; Aerocrine, Solna, Sweden) prior to VOC collection and spirometry as per manufacturer's recommendations. For VOC analyses 1-L of breath was collected across sorbent tubes packed with Carbograph 1TD/Carbograph 5TD (Markes International, Llantrisant, UK), at a flow rate of 500 mL·min^−1^ using an in-house sampler described elsewhere [4]. A background air sample was taken at every time-point by strapping the mask to a glass head and sampling 1 L of filtered air. Sorbent tubes were sealed and refrigerated immediately after sampling and analysed within 1 month. The thermal desorption-gas chromatography-mass spectrometry protocol has been published previously [5].

All VOC data files were converted to the open mzXML format prior to pre-processing. Chromatograms were screened for inclusion in the final dataset by manual appraisal and all samples were deconvolved and aligned using eRah. A hierarchical Gaussian process model was used to detect oscillating VOCs. Data were z-normalised on individual patients and compounds, and modelled as Gaussian processes with exponential covariance functions. The mean function of these Gaussian processes was then modelled using another Gaussian process, shared across patients, with zero mean and a periodic covariance (24 h period). This enables the model to account for inter-compound and inter-patient variation separately. The model was fitted using Hamiltonian Monte Carlo. Empirical *p*-values were obtained using Monte Carlo simulation from a null distribution of simulated non-rhythmic data and false discovery rates calculated. Analyses were implemented in R and Stan.

Compounds of interest were putatively identified using the National Institute of Standards and Technology library following the metabolomics standards initiative. VOCs were screened to remove common contaminants arising from the sampling equipment and any VOCs found to be rhythmic in the background samples.

Data from one patient with asthma were excluded due to technical faults with the GC-MS, leaving complete datasets for 10 healthy individuals and nine with asthma. The groups were matched for median (IQR) age (45.5 (27.5–49.3) *versus* 47.0 (26.0–49.5) years, *p*=0.92), body mass index (27.1 (23.4–30.5) *versus* 26.9 (22.3–27.2) kg·m^−2^, *p*=0.5) and male:female sex ratio (7:3 *versus* 7:2, *p*=1). All individuals with asthma were atopic with significantly lower median (IQR) forced expiratory volume in 1 s compared to healthy subjects (82.3 (73.0–89.0) *versus* 97.7 (91.7–105.3) % predicted, *p*=0.02) and median (IQR) prescribed daily ICS (equivalent to beclomethasone dipropionate) 400 (400–500) µg.

Of 76 breath samples collected (four time-points per participant), six were removed from the analysis due to errors in sampling or analytical processing. Background samples were collected at 59 time-points immediately prior to breath sampling (15 background samples were excluded due to errors in sampling or analytical processing). A mean±standard deviation of 312±45 compounds were detected in the breath samples. Once aligned and quality checked to remove contaminant compounds and deconvolution artefacts 102 VOCs were included in the Gaussian process analysis.

In the combined dataset, five VOCs were shown to be rhythmic (false discovery rate *p*<0.01). Dimethoxymethane, chlorobenzene and an unidentified VOC (*m/z* 56) showed a nadir in the morning, whereas the opposite diurnal pattern was seen for isoprene and 1-butoxy, 2-propanol (figure 1).

**FIGURE 1:**
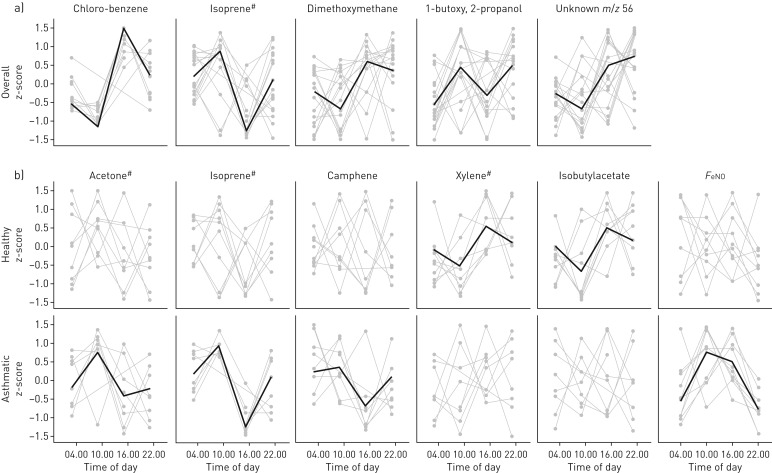
Circadian analysis of a) the pooled dataset of 19 participants for the compounds that had a false discovery rate *q*<0.05 after the Gaussian process analysis was applied and b) compounds found to be rhythmic in either the asthmatic or healthy cohort in a sub group analysis. *Z*-score values are shown in light grey with the fitted rhythm overlaid. All compounds were identified as rhythmic with a *p*-value <0.05 using the Gaussian process analysis. #: compounds were identified to metabolomics standards initiative level 1, otherwise compounds were identified to level 2. *F*_eNO_: exhaled nitric oxide fraction.

Four VOCs were rhythmic with *p*<0.05 but not significant after false discovery rate correction; acetone and 1-butanol were highest at 10:00 h and lowest at 16:00 h while xylene and phenol showed the reverse pattern.

A secondary analysis of the data was performed to investigate the rhythmic nature of VOCs associated with asthma. Camphene was annotated as being rhythmic only in asthma (*p*<0.05), with a peak at 10:00 h and a nadir at 16:00 h. Acetone and isoprene (both rhythmic in the combined analysis) were also found to be rhythmic in the asthma group alone. Two compounds were shown to be rhythmic in the healthy group but not the asthmatic group (xylene and isobutylacetate). Both demonstrated rhythms in anti-phase with VOCs found to be rhythmic in asthma only, namely a peak at 16:00 h and a trough at 10:00 h.

A rhythmic cycle for *F*_eNO_ (*p*<0.05) was detected only in the asthmatic group, with a peak at 10:00 h and nadir overnight. The median (IQR) *F*_eNO_ was 37.5 (18.3–78.0) ppb at 10:00 h and 25.5 (14.7–56.6) ppb at 04:00 h (figure 1).

We have demonstrated that there is rhythmic variability in a proportion of exhaled VOCs over 24 h. Furthermore, when comparing asthmatic to healthy breath differential patterns of VOC release were observed. Acetone is the most abundant VOC in breath and has been previously linked to asthma [6, 7]. Changes in the level of acetone overnight in this study replicate findings by King *et al.* [8]. Isoprene, the next most abundant VOC in breath, has also been linked to asthma [6, 9, 10]. Similar to acetone the changes observed in the levels of isoprene agree with previous work [8, 11]. For both VOCs, this study provides insight into the diurnal pattern of expression, adding to the nocturnal profiling detailed in the literature.

Camphene and xylene have been included in models to distinguish asthma from healthy controls [4, 12] where they were shown to be reduced in the asthmatic cohort. Camphene has also been shown to inhibit the release of nitric oxide in stressed rat macrophages [13]. All other VOCs shown to be rhythmic in this work have previously been found in breath and have been linked to a variety of diseases.

We have also shown that *F*_eNO_ demonstrates a strong circadian rhythm in asthma with lower levels detected during the night than during the day. *F*_eNO_ is used in diagnostic asthma algorithms with cut-offs varying between 25 ppb [14] and 35–40 ppb [15]. We found the diurnal *F*_eNO_ variability straddled these cut-offs and it is crucial that larger studies validate our findings, which may impact on diagnostic recommendations.

In addition to the clinical implications, this work demonstrates that time of day is an important parameter to consider when undertaking VOC sampling, especially in untargeted hypothesis-generating studies.

## Shareable PDF

10.1183/13993003.01068-2019.Shareable1 This one-page PDF can be shared freely online.
Shareable PDF ERJ-01068-2019.Shareable
