# Supplementary material for: Circadian rhythm of exhaled biomarkers in health and asthma
Source: Eur Respir J. 2019 Oct 17;54(4):1901068. doi: 10.1183/13993003.01068-2019 (PMC6796150; doi:10.1183/13993003.01068-2019)
Supplement: Supplementary file 1 [file ERJ-01068-2019.Shareable.pdf]

AGORA
RESEARCH LETTER

SHAREABLE PDF

Circadian rhythm of exhaled biomarkers in health and asthma

Maxim Wilkinson[1], Robert Maidstone[2,3,4], Andrew Loudon[5], John Blaikley[1,6], Iain R. White[1,7], Dave Singh[1,8], David W. Ray[2,3], Royston Goodacre[9,10], Stephen J. Fowler[1,6] and Hannah J. Durrington[1]

Affiliations: [1]Division of Infection, Immunity and Respiratory Medicine, School of Biological Sciences, Faculty of Biology, Medicine and Health, University of Manchester, Manchester, UK. [2]NIHR Oxford Biomedical Research Centre, John Radcliffe Hospital, Oxford, UK. [3]Oxford Centre for Diabetes, Endocrinology and Metabolism, University of Oxford, Oxford, UK. [4]Division of Informatics, Imaging and Data Sciences, School of Biological Sciences, Faculty of Biology, Medicine and Health, University of Manchester, Manchester, UK. [5]Division of Diabetes, Endocrinology and Gastroenterology, School of Medical Sciences, Faculty of Biology, Medicine and Health, University of Manchester, Manchester, UK. [6]Manchester Academic Health Science Centre and NIHR Biomedical Research Centre, Manchester University Hospitals NHS Foundation Trust, Manchester, UK. [7]Laboratory for Environmental and Life Sciences, University of Nova Gorica, Nova Gorica, Slovenia. [8]Medicines Evaluation Unit (MEU), Langley Building, Manchester University Hospitals NHS Foundation Trust, Manchester, UK. [9]School of Chemistry, Manchester Institute of Biotechnology, The University of Manchester, Manchester, UK. [10]Dept of Biochemistry, Institute of Integrative Biology, University of Liverpool, Liverpool, UK.

Correspondence: Hannah J. Durrington, Division of Infection, Immunity and Respiratory Medicine, School of Biological Sciences, Faculty of Biology, Medicine and Health, The University of Manchester, Manchester, M13 9PT, UK. E-mail: hannah.durrington@manchester.ac.uk

@ERSpublications
Exhaled volatile chemicals and fractional exhaled nitric oxide oscillate over 24 h, highlighting the importance of time of day in diagnostic sampling and suggesting potential applications for chronotyping. http://bit.ly/2YZGXbH

Cite this article as: Wilkinson M, Maidstone R, Loudon A, *et al*. Circadian rhythm of exhaled biomarkers in health and asthma. *Eur Respir J* 2019; 54: 1901068 [https://doi.org/10.1183/13993003.01068-2019].

This single-page version can be shared freely online.

To the Editor:

Circadian rhythms regulate and reflect many biological processes. Investigating circadian variability in biomarkers is important since the diurnal variability of any potential biomarker must be quantified and controlled in research and clinical practice. Time of day is particularly important in inflammatory diseases such as asthma, which are linked to exaggerated circadian rhythms. Airway narrowing in asthma is greatest at around 04:00 h and coincides with an increase in symptoms; asthma deaths are also more likely to occur at this time [1, 2]. Likewise eosinophilic airway inflammation peaks in the morning, with clinical implications for biomarker-guided steroid therapy [3].

Copyright ©ERS 2019 This article is open access and distributed under the terms of the Creative Commons Attribution Licence 4.0.
